# Models of development sociology in the age of artificial intelligence: a PRISMA-guided scoping review of global research trends (2020–2025)

**DOI:** 10.3389/frma.2026.1879245

**Published:** 2026-07-16

**Authors:** Ahmed Mohi Khalaf Sakr, Maan Yousef Al Saati, Aicha Ettaieb, Asmaa Ahmed

**Affiliations:** 1 Department of Sociology, College of Arts and Sciences, Umm Al Quwain University, Umm Al Quwain, United Arab Emirates; 2 Department of Sociology, Faculty of Arts, Minia University, Minya, Egypt; 3 College of Arts and Sciences, Umm Al Quwain University, Umm Al Quwain, United Arab Emirates; 4 Department of Sociology, Ajman University, Ajman, United Arab Emirates; 5 Department of Sociology and Social Work, University of Fujairah, Fujairah, United Arab Emirates

**Keywords:** artificial intelligence, development sociology, Global Trends, PRISMA, scoping review

## Abstract

**Introduction:**

This study presents a PRISMA-guided scoping review of global research trends in the sociology of development during the period 2020–2025, with particular emphasis on the emerging intersection between development studies and Artificial Intelligence (AI).

**Methods:**

Thirty-five peer-reviewed studies were systematically selected from the Scopus, SAGE, Wiley, and JSTOR databases. A systematic search strategy and qualitative content analysis were employed to identify the dominant thematic areas, theoretical frameworks, and methodological approaches in the reviewed literature.

**Results:**

Social development emerged as the most prominent research theme, accounting for 28.6% of the reviewed studies, followed by social justice (17.1%), social policy (14.3%), and sustainable development (11.4%). Explicit engagement with AI-related perspectives remained limited. Qualitative approaches constituted the dominant methodological paradigm (60%), with case studies and participatory research being the most frequently employed methods, whereas quantitative and computational approaches remained comparatively limited.

**Discussion:**

The reviewed literature revealed limited theoretical and methodological integration of AI within development sociology, highlighting the need for innovative interdisciplinary approaches. The study concludes that the sociology of development is moving toward greater theoretical pluralism and methodological diversity; however, stronger integration with digital transformation and AI-enabled development research remains necessary.

## 1 Introduction

Development Sociology is a prominent branch of sociology concerned with understanding the social processes, institutional dynamics, and structural inequalities that shape development routes across societal contexts. While traditional approaches have focused on modernization, dependency, and social transformation, contemporary Development Sociology expanded its analytical scope to include the effects of digitalization, technological innovation, and evolving governance that influence development outcomes in the twenty-first century (McMichael, 2017; Horner and Hulme, 2019). Recent studies identify Artificial AI as a critical driver of socio-economic transformation, influencing labor markets, public administration, social inclusion, and sustainable development agendas worldwide (Vinuesa et al., 2020; UNESCO, 2021; OECD, 2022; Dwivedi et al., 2023).

Development Sociology is currently undergoing a significant epistemological and structural transformation under the accelerating pressures of globalization, digitalization, and rapid technological change. Among these transformative forces, AI has developed not merely as an external technological innovation, but as an increasingly constitutive element of contemporary social systems, reshaping governance systems, labor structures, inequality patterns, and development trajectories in complex and often uneven ways.

This transformation signals a shift beyond traditional development paradigms, as classical sociological models struggle to adequately capture the speed, scale, and algorithmically mediated nature of contemporary social change. AI-driven systems are now central to data production processes, policy design, and institutional decision-making, yet they simultaneously intensify structural inequalities through digital divides, opaque algorithmic governance, and asymmetrical access to technological infrastructures. These contradictions highlight a growing tension between technological acceleration and normative concerns of social justice, equity, and inclusion.

International organizations and policy institutions have acknowledged that AI is reshaping the conceptual foundations of human development. Reports from UNESCO (2021) and OECD (2022) emphasize the need for ethical, inclusive, and sustainable governance frameworks capable of regulating AI's expanding role in social and economic life. However, these normative discourses often remain disconnected from systematic sociological analysis, particularly regarding how AI is reshaping the internal epistemology of Development Sociology itself.

Despite the great growth of interdisciplinary literature on AI and development, current research is still fragmented and conceptually uneven. Most studies either deals with AI as a technological variable within development outcomes or focus on macro-level policy implications, without sufficiently interrogating its impact on theoretical paradigms, methodological designs, and knowledge production systems within Development Sociology. This reveals a critical gap in literature: the absence of a systematic, globally oriented synthesis that maps how Development Sociology has evolved under the influence of AI during the period 2020–2025.

From a knowledge construction perspective, scientific progress depends on the systematic accumulation, organization, and critical interrogation of existing literature. Literature reviews are not merely descriptive exercises but constitute epistemological tools that reveal disciplinary boundaries, intellectual trajectories, and structural gaps. In this sense, mapping global research trends is essential not only for identifying thematic patterns but also for understanding how a discipline defines and redefines its own analytical limits.

Accordingly, this study addresses this gap through conducting a PRISMA-guided (Preferred Reporting Items for Systematic Reviews and Meta-Analyses) scoping review of global Development Sociology research (2020–2025). It thoroughly examines thematic orientations, theoretical frameworks, and methodological approaches, with particular attention to the underexplored intersection between AI and Development Sociology. In doing so, the study seeks to contribute to a more reflexive and critically informed understanding of how development knowledge is being reshaped in the age of intelligent systems.

## 2 Research objectives

2.1 Identify dominant themes in Global Development Sociology (2020–2025).
2.2 Analyze theoretical frameworks applied in the literature.
2.3 Examine methodological approaches used in empirical studies.
2.4 Assess the extent of AI integration in development sociology research.

## 3 Research questions

3.1 What are the dominant thematic areas in Global Development Sociology (2020–2025)?
3.2 Which theoretical frameworks are most frequently used?
3.3 What methodological approaches dominate the field?
3.4 How is AI represented in Development Sociology research?

## 4 Methodology

The study adopts a PRISMA-guided scoping review, combined with qualitative content analysis, to systematically map global research trends

### 4.1 Scope and eligibility criteria

The scope of the review was identified to capture recent and relevant academic contributions at the intersection of Global Development Sociology as follows, the literature search followed PRISMA guidelines and included the following steps:

#### 4.1.1 Databases

Multiple major academic databases, including Scopus, SAGE Journals, Wiley Online library, and JSTOR were used to conduct a comprehensive literature search. These platforms were selected for their wide coverage of peer-reviewed works in the field of development sociology; Scopus was selected for being one of the most comprehensive indexing platforms for measuring intellectual output and review studies and considered the primary database for its extensive coverage of international peer-reviewed journals in the social sciences, humanities, and technology-related disciplines. SAGE was included for its robust coverage of sociology, development studies, social policy, and interdisciplinary social research. Wiley was added to the study due to its large collection of journals relevant to the research area. GESTOR was also included to ensure access to foundational and theoretical literature in sociology and development studies, including influential conceptual contributions that might not be comprehensively indexed in citation databases.

#### 4.1.2 Boolean search string

Boolean keyword combinations were also employed to identify relevant literature, including “Development Sociology” AND “Global Trends,” “Social Development” AND “AI,” “Sustainable Development” AND “Sociology,” and “Participatory Development” AND “Governance.”

#### 4.1.3 Time frame

The study focuses on publications within the period from 2020 to 2025, a timeframe that captures the most recent and relevant developments in Development Sociology in the context of rapid global digital transformation and the growing influence of AI on social and developmental processes.

#### 4.1.4 Inclusion criteria

The sources were selected based on well-defined inclusion criteria to ensure reliability and significance of the literature. Peer-reviewed journal articles, conference proceedings and book chapters related were taken into account. The study focused on works published between 2020 and 2025 to guarantee employing the latest articles in the field of development sociology. Furthermore, the selected works shall be only about themes related to development sociology. And all these works were required to be indexed in recognized international databases.

#### 4.1.5 Exclusion criteria

To maintain the scholarly objectivity and relevance of the review, a clear exclusion criterion was followed in this work. Firstly, non-academic publications were omitted to ensure that only reliable and research-based sources were employed in the study. In addition, Opinion articles and editorials were also excluded, as they lack systematic analysis. The study also excluded the studies outside sociology or development fields to ensure that the chosen literature fits the study's analytical focus.

### 4.2 Search strategy and screening process

**Figure 1:**
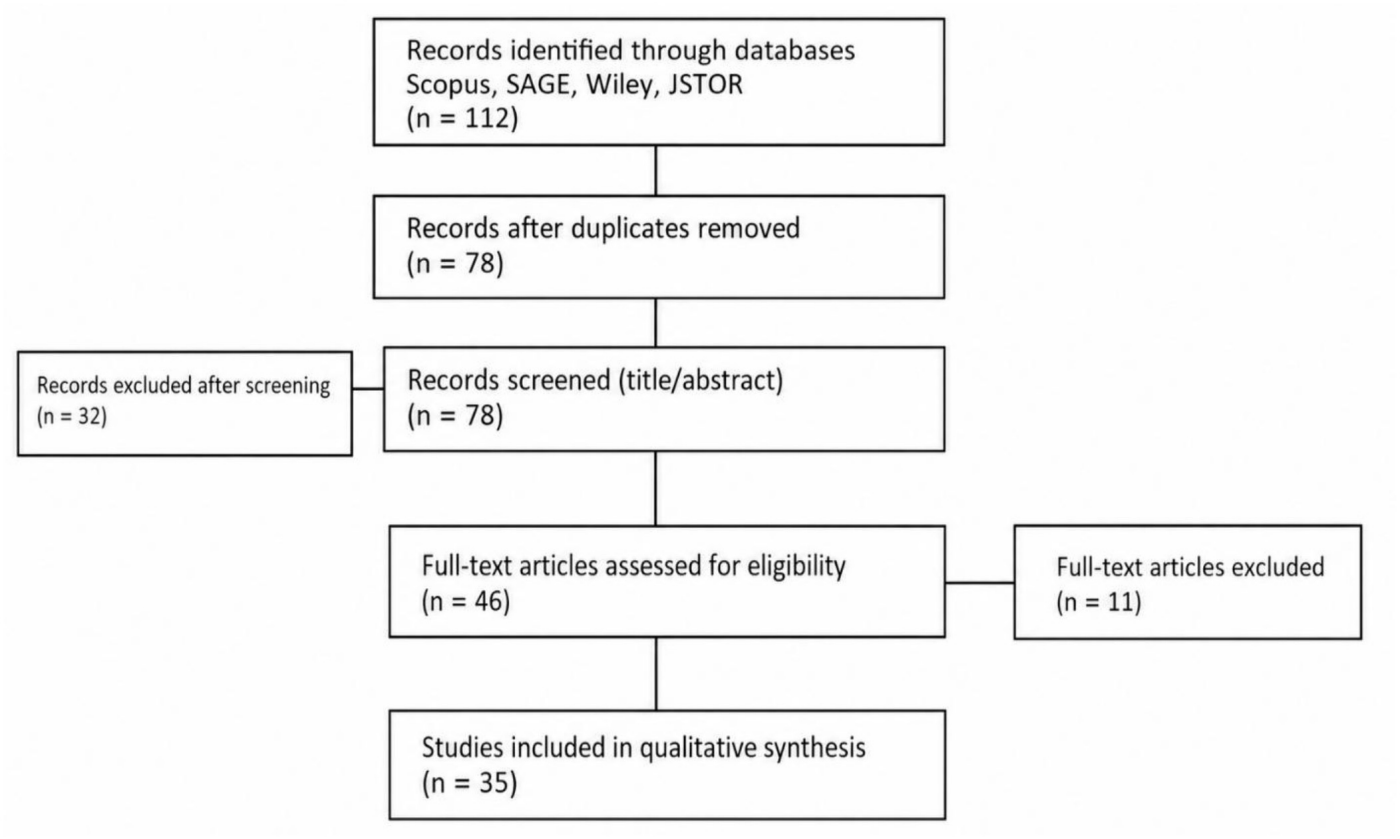
PRISMA flow diagram of study selection process. Reasons for exclusion included irrelevance to Development Sociology, lack of empirical focus, and non-indexed publications.

A systematic search of the literature across Scopus, SAGE, Wiley, and JSTOR databases initially yielded 112 records relevant to the scope of this study. After the removal of duplicates and the exclusion of studies not meeting the predefined eligibility criteria, 78 records were retained for title and abstract screening. Subsequently, 46 full-text articles were assessed for detailed eligibility based on relevance to Development Sociology and the study's thematic focus. Following this rigorous screening process, 35 studies met all inclusion criteria and were ultimately included in the final qualitative synthesis. These studies constitute the analytical corpus of the present PRISMA-guided scoping review. The study selection process followed PRISMA guidelines, including participant identification, screening, eligibility assessment, and final inclusion stages. Detailed filtering steps and the number of exclusions are shown in Figure 1.

### 4.3 Data analysis

The data were analyzed using qualitative content analysis following a systematic and iterative analytical procedure. The analysis began with an open thematic coding process to identify recurring concepts and patterns across the selected studies. This was followed by axial coding to refine and cluster themes into higher-order analytical categories. Subsequently, theoretical classification was conducted to map the dominant conceptual frameworks underpinning Development Sociology research, allowing for a structured comparison of theoretical orientations across studies.

In addition, methodological mapping was employed to systematically examine research designs, data collection techniques, and analytical strategies used in the literature. This process enabled the identification of methodological tendencies, including the predominance of qualitative approaches and the limited integration of quantitative and computational methods. The combined analytical strategy ensured a comprehensive and multi-layered interpretation of the dataset, enhancing the reliability and analytical depth of the scoping review. Data were analyzed using qualitative content analysis focusing on, Thematic coding, Theoretical classification, and Methodological mapping.

To ensure methodological transparency and facilitate comparative analysis, the selected studies were subjected to predefined criteria, including publication year (2020–2025), source type, country of publication, and disciplinary classification. These classification parameters served as the basis for the descriptive presentation of the literature and informed subsequent analytical comparisons. Furthermore, references of the selected studies were listed in the following table to improve traceability and and facilitate readers' understanding of the connections between the bibliographic dataset and the findings presented in this review (Table 1).

**Table 1:** Bibliometric distribution of selected peer-reviewed journals, book series, and conference outputs in development sociology (2020–2025).

Category	Source type	Journal/source	Country	References	No. of studies
Humanities and interdisciplinary studies	Journal	Journal of Advanced Academic Studies	Bangladesh	Sundram, 2020	1
Humanities and interdisciplinary studies	Journal	Kurdish Studies	Hong Kong	Sakr et al., 2024a	1
Humanities and interdisciplinary studies	Journal	Journal of Ecohumanism	UK	Altaany et al., 2024	1
Humanities and interdisciplinary studies	Journal	Open Philosophy	Germany	Fourie and Sands, 2022	1
Humanities and interdisciplinary studies	Book Series	IntechOpen	UK	Burns, 2022	1
Social sciences	Journal	Asian Social Science	Canada	Mohamed et al., 2020	1
Social sciences	Journal	Swedish Institute for Social Research	Sweden	Sirén, 2022	1
Social sciences	Conference Proceedings	UNDESA Virtual Expert Group Meeting	UNDESA	Millard, 2020	1
Development sociology (specialized journals)	Journal	Social Development Issues	USA	Such as, Gray (2024) and Amoah (2020)	12
Development sociology (specialized journals)	Journal	Sociology of Development	USA	Rademacher and Pumar, 2023	1
Development sociology (specialized journals)	Journal	Canadian Planning and Policy	Canada	Bautista et al., 2024	1
Development sociology (specialized journals)	Journal	Journal of Infrastructure, Policy and Development	USA	Sakr et al., 2024b	1
Development sociology (specialized journals)	Journal	Community Development Journal	UK	Such as, Galán-Guevara (2024) and Hlela (2022)	12
**Total**					**35**

Classification is based on source type (peer-reviewed journals, academic book series, and conference proceedings) following PRISMA-guided inclusion criteria. All entries represent selected studies included in the final analytical sample (*n* = 35). Bold values indicate the total frequencies for each category.

## 5 The literature of the study

The reviewed studies were categorized into nine thematic categories through an inductive analysis process, rather than being predetermined. Initially, open coding was applied to the full texts of the selected studies to identify recurring themes, conceptual concerns, and core areas of focus. Similar codes were then grouped using pivotal coding to generate broader thematic domains. Through repeated discussions and comprehensive comparisons of the datasets, nine main categories emerged, reflecting prevailing research trends in the contemporary Development Sociology literature. These can be presented as follows:

### 5.1 Social development

The paper of Altaany et al. (2024) addressed Social legislation, policies, and laws related to individuals' welfare and responsibility, including education, health, employment, and civil rights. Using a systematic literature review, the work could explore historical developments, theoretical conceptualizations, and empirical reviews of cross-national findings on the topic. The analysis highlighted that strong social legislation has positive impacts on various aspects of welfare. Moreover, universal health care laws are expected to reduce health disparities, and compulsory education laws improve literacy and social mobility. Similarly, Labor legislation improves employment satisfaction, leading to reduced poverty rates, and contributes to economic security. Civil rights laws work against discrimination; reinforce the unity of society and encourage equality. However, challenges such as insufficient funding, corruption, and cultural barriers affect the program implementation, especially in low- and middle-income countries. The paper recommends improving measures relating to enforcement, popularization of existing legislation, means of financing, and legislation's tailoring to regional specifics. Addressing these factors makes social legislation a powerful lever toward social development and the building of a society based on justice.

Another leading article of sociological studies on social development is the paper of Hong (2024), which introduced a new model focused on promoting human-centered business practices. The study aimed to show how programs and practices that foster psychological self-reliance can improve employment opportunities and promote social integration for low-income individuals. This, in turn, contributes to financial independence, quality of life, increased job opportunities, and overall higher levels of human development. The research employed a mixed-methods approach, combining both quantitative and qualitative analyses. It emphasized the examination and assessment of real-world development programs, particularly within American society, such as the Summer Youth Training Program and Transforming the Impossible into Possible program.

The research of Sakr et al. (2024a) explored the acceptance levels of digital work opportunities within a youth demographic, aiming to discern the underlying factors influencing their embrace or refusal of such opportunities. To accomplish these objectives, a purposive random sample comprising 177 unemployed individuals aged between 15 and 30, representing all seven emirates, was selected. The study employed a methodology rooted in sample social survey techniques. Results indicate a prevailing acceptance of digital work opportunities within the youth culture of the UAE. A key catalyst for this acceptance is identified as the acknowledgment that digital technology has become the language of the current era, as expressed by 61.4% of the study sample. Conversely, reasons for rejecting digital work culture among certain participants' center around difficulty and lack of training, cited by 50% of those who declined digital work opportunities. This study sheds light on the dynamic interplay between youth and evolving work landscapes in the context of development.

Midgley's (2024) paper aimed to explore the concepts of change and progress in the context of social development, with an emphasis on technological progress. It investigated how these concepts affect the lives of individuals and communities, and how modern technology plays a dual role in social development, in terms of providing new opportunities or posing challenges that need to be addressed. To answer these questions and meet the goals, the paper adopted an analytical approach based on a review of existing literature and previous studies, in addition to providing practical examples from global social development works with a special focus on practices in India.

Gray's (2024) paper aimed to analyze the progression of social development theory and its implementation in practice, highlighting the challenges encountered and critiquing the influence of Neoliberalism on social development. The study introduced a critical model of social development practice taking into account the structural, political, cultural, and environmental dimensions of South Africa. The paper adopted a critical analytical methodology, reviewing previous literature and theoretical models, and analyzing social policies in South Africa and their impact on development practices.

Similarly, Lohman et al. (2023) aimed to explore the importance of learning from the history of community development and how historical knowledge can contribute to improving current practices and help avoid repeating past errors. Their study explored diverse experiences of community work including Bangladesh, India, and Ireland. The paper adopted an analytical approach based on a review of historical literature and previous studies in the field of community development, providing examples of contemporary community development practices.

Rademacher and Pumar's (2023) study explored the evolution of sociology in the field of development, focusing on how the sociological understanding of development has diversified and expanded through new theoretical and methodological approaches. It also highlighted the importance of comprehensive dialogue and constructive criticism in shaping sociological debates, and how this can impact future policies and practices. The paper relied on an analysis of existing literature and scholarly discussions held within the framework of the Development Sociology Department of the American Sociological Association.

Sundram's (2020) research paper aimed to provide a thorough perspective on how to promote social development in children through collaboration between family, school, and community. This approach focuses on social development starting from early childhood. The paper relied on a review of previous studies related to social development, in addition to providing practical guidance for parents and teachers on how to help children build their social skills, along with an overview of the stages of social development across different ages. Similarly, Mohamed et al.'s (2020) research paper supports this issue, as they aimed to provide a critical analysis of the concept of social development, including its features, definitions, dimensions, and frameworks. Their study aimed to highlight shortcomings in current frameworks and propose improvements. The authors adopted content analysis approach through reviewing and analyzing development-related sources, including books, articles, conferences, and reports.

Sobantu's (2020) research paper highlighted the relationship between adequate housing and social development, clarifying how satisfactory housing contributes to improving the lives of individuals and communities by promoting economic growth, reducing poverty and inequality, as a tool for promoting social development. The paper relied on existing literature on housing and social development, using prior research and real-world experiences in South Africa aiming to highlight the challenges and potential opportunities in this field.

### 5.2 Social movements

One of the global sociological works on social movements was the research by Kiely et al. (2024), which aimed to explore the relationship between community development and the prison state. This work relies heavily on the use of prisons and detention as a means of controlling individuals and communities. This includes the expansion of punishment systems, having more prisons, and using coercive force against individuals. The paper focused on how penal systems impact community development and highlighting the role of active social movements in facing these systems in Canada and India, such as the Surrey Union of Drug Users, which confronts repressive drug policies and provides support to individuals affected by these policies. These movements contribute to focusing on development more than punishment, aiming to adopt a developmental approach empowering those targeted by these policies. The paper relied on a critical analytical approach, analyzing a group of articles and reviews on the subject.

Likewise, Ruggiero's (2024) research aimed to discuss the impact of punishment on individuals in different societies, and how abolitionist social movements, or anti-slavery movements, which seek to abolish a particular system of punishments or practices that are considered unjust or inhumane in addressing crimes and violations through more humane and effective alternatives, can promote social justice by developing new alternatives. The paper employed an analytical critical approach, as previous literature and studies related to punishment and societies were used and analyzed.

### 5.3 Social justice

The study by Pankaj and Yadav (2023) is one of the leading global sociological works focusing on social justice. This work aimed to explore the ethical challenges faced by community development practitioners in India during COVID-19 pandemic, focusing on their daily experiences working with vulnerable communities such as Dalits, women, and migrant workers to achieve social justice. The research used a qualitative approach, represented by narrative in-person and telephone interviews.

Khatoon and Kumar (2023) also explored the ethical challenges faced by community development researchers during their research with a derecognized tribal community in Bihar, India. The study focused on the importance of reflexivity and ethical practices in community research for promoting social justice. The research relied on a qualitative approach, where group discussions and interviews were conducted with a number of community development researchers in five villages in the Arya district of Bihar, as well as community members, to understand their experiences and challenges.

Similarly, the research paper by Mohan (2022) studied the relationship between social justice and social development, concentrating on issues of inequality and racial discrimination and their impact on democratic values. It aimed to provide insights into how to enhance social justice in a complex global context, with particular attention to American society, which, from the researcher's point of view, was deeply divided. The research relied on an analytical and critical approach, where texts, ideas, and literature related to social justice and development are analyzed.

The study by Fourie and Sands (2022) analyzed the essential flaws in Western democracy, particularly in the context of economic and social inequality. It also explored how the competitive nature of Western democracy, known as winner-takes-all, leads to a reduction in political choices and the neglect of social and economic issues, ultimately hindering the pursuit of social justice.

The article called for a reconsideration of how to improve democratic governance to more effectively address these issues. The paper adopted a critical analysis of ideas and theories related to democracy, drawing on philosophical and social approaches. An analytical approach was used to explore key concepts such as choice, freedom, and democracy, and their influence on addressing inequality.

Also, the research paper by Olonisakin and Idemudia (2021) aimed to study the relationship between social dominance, intolerance toward outgroups, and the influence of spiritual values, transcendence values, on enhancing tolerance and openness to diversity. The research relied on a social survey method with a sample of 100 people from different ethnics in Nigeria, through a questionnaire designed to measure variables related to social dominance orientations, and intolerance toward outgroups.

### 5.4 Social policy

One of the important global sociological works addressing social policy is Johnson's (2024) paper which aimed to develop a conceptual framework for understanding social policies in modern welfare states that serve both preventive and productive roles. It explored developmental principles and how they can be integrated into welfare states literature, with particular reference to countries in the Global North, such as Scandinavia. The study also explored how these concepts might be extended to the Global South through insights drawn from Northern welfare models. Therefore, the paper relied on an analytical approach based on a literature review, combining development-related ideas from the Global North.

Niigaaniin et al. (2023) also evaluate the Social Assistance Program, with particular attention to the Anishinabek experience in Ontario, aiming to make it more effective and relevant to clients. The paper focuses on incorporating Indigenous cultural perspectives into the design of social services. This study relied on a participatory research approach, where focus group sessions were conducted involving both clients and community members. The sample included 292 clients who benefited from social services and participated in focus group sessions.

Cheng and Kumar (2022) also studied the social networks and experiences of resettling Chinese refugees in the United Kingdom, focusing on how these connections impact their wellbeing in the new society, and how to improve social support and social policies targeting this group in the United Kingdom.

The research employed participatory activities such as social mapping and card sorting to collect data from participants, involving Chinese refugees in in Glasgow and Scotland, and staff from the Chinese Community Development Organization (CCDP), to collect the needed data about social ties and levels of trust and exchange among refugees.

The book by Sirén (2022), which contains three studies, also aimed to analyze factors influencing the reform of social policies in low- and middle-income countries, highlighting the role of social protection as a key element in achieving the Sustainable Development Goals. The first study investigated the reasons of changes in social spending across 46 newly democratic countries, paying special attention to the role of party politics, using data from 1995 to 2015 to assess its impact on improving social policies. The second study explored the mechanisms that facilitate progress toward expanding social policy, concentrating on policies related to health care reform in Bolivia. The third study evaluated the outcomes of social policy through analyzing how government cash transfer systems mitigate the impact of economic growth on both absolute and relative poverty. Thus, the book employed in its three studies on a multi-methodology including, quantitative analysis using data from 46 newly democratic countries between 1995 and 2015, and In-depth case studies such as Bolivia and Luxembourg to understand different contexts and their impact on social policies.

Bäckman's (2021) research paper aimed to raise awareness about global inequality, focusing on poverty which leads to social disability. It also explored that social policies lead to social justice, solidarity, and equal values, while employing advanced technology to support these values in society. The study used an analytical approach employing existing literature and studies, with a focus on how technology and social changes affect social policies.

### 5.5 Participatory development

Bautista et al. (2024)) is one of the global sociological works that focused on participatory development. This study aimed to provide a comprehensive vision on improving urban social planning through participatory methods by reinforcing collective capacities, thus contributing to promoting more just and inclusive societies. It also highlighted the importance of focusing on integrating community institutions into community planning and enhancing understanding of how their participation impacts meeting and planning for neighborhood needs. The research adopted the community-engaged research methodology, which included data collection through the analysis of demographic, social, and economic data from neighborhoods, in addition to conducting interviews with residents to collect qualitative data.

Similarly, Pandeya et al.'s (2022) conducted a study to explore the impact of social mobilization on local governance performance in rural communities in Nepal, with the aim of enhancing community participation and empowerment through these processes. The study relied on a qualitative approach using multiple tools such as interviews, observations, and focus group discussions. A random sample included 22 stakeholders, in addition to 31 individuals in the Godamshor district and 44 individuals in the Erko district of Nepal. Hlela's (2022) study also aimed to identify adult learning experiences related to participatory development in a rural African village in South Africa, focusing on the importance of participation in the learning process. The study used a qualitative approach, conducting case studies and interviews with local community members to understand their experiences and views on participatory learning.

The research of McGregor and Scandrett (2022) explored the role of community development in confronting climate emergencies, particularly by highlighting the experiences of marginalized communities, such as Palestinian women and indigenous peoples in Canada. This study focused on how climate change affects these communities, and how to engage them to confront these climate fluctuations within the framework of achieving social development. The paper employed qualitative research approaches, including interviews and case studies.

### 5.6 Community empowerment

Leading the field of global sociological works on community empowerment is the paper by Raj et al. (2023), which aimed to highlight the relationship between community development initiatives and health policies to promote health equity, community empowerment, and reduce health gaps. This study focused on the experiences of several countries, such as the United Kingdom, Italy, and Canada, highlighting the importance of community empowerment as an effective tool for improving health and reducing health gaps. It also referenced relevant theories and prior research work. The research used a literature review and policy analysis to highlight the challenges and opportunities in integrating community development with public health in these countries. Bolton (2022) also sought to provide a critical framework on community development related to displacement and housing justice, concentrating on the importance of anti-displacement policies in countering the effects of gentrification—a process in wich low- or middle-income areas begin to attract higher-income residents, leading to improvements in urban areas but also potentially causing social and economic problems for indigenous populations. The study focused on the United States, specifically Pasadena, California, and relied on participant observation and document analysis, with data analyzed through direct observation at community development conferences such as those of the Christian Community Development Association (CCDA), and reviewing documents related to the research topic.

### 5.7 Sustainable development

One if the leading works on sustainable development is the research of Sakr et al. (2024b) which aims to explore the acceptance degree of digital and sustainable work culture among youth in the Emirati society. It revealed the relationship between gender and educational status as sociodemographic factors among youth in the study sample and their level of acceptance of digital and sustainable work culture. Furthermore, the study aims to identify prospective trends in digital work culture among young individuals in Emirati society. Due to the nature of descriptive research, it employed the sample social survey approach. The field study primarily utilized a quantitative tool for data collection, namely the digital questionnaire. This questionnaire was administered to a random sample comprising young individuals actively seeking employment opportunities, unemployed individuals, or those new to the labor market. The questionnaire covered participants aged between 15 to 35 years, with a total of 184 individuals. The sample covered all youth categories in Emirati society, considering demographic factors such as gender, place of residence, and educational status. The findings indicated that an overwhelming majority of young individuals in the study sample 97.8% have no obstacles to accepting job opportunities that necessitate digital and technological skills. Moreover, the study uncovered a direct and statistically significant relation between gender and the level of acceptance of digital work culture, favoring females. This implies that females are more inclined to accept digital job opportunities compared to males. Additionally, the results highlighted a positive and statistically significant relation between both educational status and the level of acceptance of digital work culture. In other words, individuals with higher levels of education demonstrate a greater interest in digital job opportunities. Utilizing Stepwise Regression, the study also made predictions about the spread of future digital work culture in the United Arab Emirates based on the variable of education.

Among global sociological works addressing sustainable development was the research by Galán-Guevara (2024), which aimed to provide a comprehensive understanding of the changes occurring in the community of San Cristóbal de la Laguna in Mexico. The research studied how these changes could impact the community's cultural and environmental identity, thereby negatively impacting the progress of sustainable development. It focused on local livelihoods, and traditional economic practices, and their impact on social and environmental relations. The study employed a qualitative approach, using direct observation, participation, in-depth individual interviews, and participatory workshops for a random sample representing 3% of households, and 18 in-depth interviews.

Another important global sociological work on sustainable development is the research paper by Sharma (2024), offered a comprehensive vision on how sustainability cab be achieved by focusing on the social and cultural dimensions, and how the three elements, people, culture, and mindset affect the progress toward sustainability. This was to enhance the general understanding of the importance in achieving sustainable development goals. The study discussed making sustainability an integral part of daily life through embedding its principles in all aspects of life. It emphasized culture as an influential factor for sustainability, emphasizing its role in shaping behaviors and values, as a means of social change, and as a link between mindset and individuals. It also highlighted the cultural challenges that may face supporting sustainability. The paper relied on conducting a comprehensive literature review to explore existing studies that support the need for and the relationship between mindset, people, and culture in achieving sustainability.

Millard (2020) also sought to clarify the close relationship between social justice and sustainable development, emphasizing the need for social policies to achieve the shared goals of both concepts. The study highlighted the impacts of the COVID-19 pandemic on social development and explored objectives for ensuring a just transition toward sustainability during times of crises. The research paper adopted a qualitative approach through analyzing data related to the impacts of the pandemic on various aspects of social development. It focused on experiences in Scandinavian countries, Sweden, Norway, Denmark, Finland, and Iceland. This model combines flexibility in the labor market and social security. It indicated that these countries are a model for achieving a balance between flexibility in the labor market and social security, which enhances the ability of individuals to adapt to economic and social changes and achieve the goals of sustainable development.

### 5.8 Human development

One good example of recent global sociological works addressing human development was the paper by Burns (2022), which aimed to explore the relationship between Amartya Sen's capabilities approach and the concept of happiness, explaining how Sen expressed the importance of this concept in the context of human development. The paper relied on a critical analysis of the literature related to the capabilities approach, comparing Sen's ideas with those of other scholars, such as Richard Layard, offering a comprehensive view of how these theories interact with each other and how they can impact our understanding of human development and well-being.

### 5.9 Social protection

Among the forefront of global sociological works that focused on social protection was the research by Amoah (2020), which examined the role of funeral insurance in promoting social protection in the Ghana's Dorimon region, with particular attention to gender dimensions. The research employed a qualitative research approach including in-depth interviews with members of the funeral insurance group. Data was collected, in the Dorimon area, from a purposive sample of male and female farmers aged between 20 and 65.

## 6 Results

### 6.1 Thematic trends and emerging gaps in development sociology research

A review of the global sociological literature on development sociology reveals a diverse range of themes and issues related to development factors. These can be classified into nine development areas, as illustrated in Table 2.

**Table 2:** Thematic Distribution of global sociological works in development sociology research during the period from 2020 to 2025.

No.	Theme	Frequency	Percentage	Representative references
1	Social development	10	28.6%	Such as, Altaany et al. (2024), Hong (2024), Sakr et al. (2024a)
2	Social justice	6	17.1%	Such as, Kiely et al. (2024), Ruggiero (2024)
3	Social policy	5	14.3%	Such as, Johnson (2024), Niigaaniin et al. (2023)
4	Sustainable development	4	11.4%	Such as, Sakr et al. (2024b), Galán-Guevara (2024)
5	Participatory development	4	11.4%	Such as, Bautista et al. (2024), Pandeya et al. (2022)
6	Social movements	2	5.7%	Such as, Kiely et al. (2024), Ruggiero (2024)
7	Community empowerment	2	5.7%	Such as, Raj et al. (2023), Bolton (2022)
8	Human development	1	2.9%	Burns, 2022
9	Social protection	1	2.9%	Amoah, 2020
**Total**		**35**	**100%**	

Bold values indicate the total frequencies for each category.

Data given in Table 2 indicate that researchers have shown interest in development topics related to social development 28.6%, social justice 17.1%, and social policy 14.3% of the total global sociological studies in development sociology research. Perhaps the forefront of interest of researchers in these issues reflects the focus of their development sociology research on human-centered issues and the pursuit of human wellbeing, core elements, core meaning of truly achieving development.

The data given in the table reveals that global researchers are moderately interested in development topics related to both participatory development and sustainable development, with percentages reaching 11.4% of the total global sociological works in development sociology research, respectively. This indicates the presence of sustainability issues and participation in development at a moderate level, contrary to what is expected from the interest in sustainable development issues in global research.

The table reveals the presence of noteworthy, though less frequently addressed, topics among researchers. These topics are related to social movements, social protection, and community empowerment, representing 5.7%, 2.9%, and 5.7% respectively of the total global sociological work in development sociology research, Additionally, the table data also point to a decline in researchers' interest in development topics and issues related to human development, representing 2.9% of the total global sociological work in development sociology research. This result is noteworthy, raising questions about why the latter has not received more attention in this body of research.

One of the most notable observations is the decline in interest in topics such as digitalization and its relationship to development, despite their growing global relevance. There is a lack of research addressing topics related to digitalization and the effects of AI on achieving development, including the concept of open development. This concept, understood as an evolving theoretical and epistemological framework, depends on the idea that successful development models can be transmitted to developing countries via digital networks, thereby fostering economic growth. It is grounded in the view that digitalization, particularly through mechanisms tied to intellectual property, offers an effective pathway for addressing the challenges faced by developing economies.

This indicates that Social Development remains dominant, while AI-related and digital sociology themes are still underrepresented. Despite the growing global importance of AI-driven development initiatives, none of the studies categorized under social protection, community empowerment, or human development explicitly included AI as a conceptual framework or empirical focus. This finding reveals a significant gap in the sociology of development literature.

### 6.2 Theoretical frameworks trends in global sociological studies in development sociology research

It can be noticed that all global sociological works in the development sociology research have employed theoretical approaches relevant to their topics. This reflects researchers' interest in the role of theory in explaining developmental issues and themes related to their research. Based on this, global sociological studies in development sociology research can generally be categorized into just two main types based on adopted theoretical trends:

- The first type includes sociological studies where authors relied on a single theoretical approach that aligns with the nature of their research topics, such as studies by Sundram (2020), Mohamed et al. (2020), and Amoah (2020).
- The second type includes sociological works in which authors adopted multiple theoretical approaches, through combining more than one theoretical framework, such as studies by Gray (2024), Midgley (2024), Hong (2024), and Johnson (2024).

Overall, global sociological studies in development sociology have diversified their use of various modern, contemporary, and traditional theoretical frameworks, which are effective in analyzing many contemporary development issues. Among these modern and contemporary frameworks addressed in global sociological works referenced in this article are, Theory of Social Justice, Social Development Theory, Theory of Collective Capabilities, Theory of Sustainable Development, Development Critique Theory, Theory of Social Welfare, Theory of Social Protection, Neoliberalism Critique Theory, and Theory of Social Investment. In general, the most important theoretical trends addressed by global sociological works can be identified in the (Table 3).

**Table 3:** Theoretical Frameworks addressed by global sociological works in development sociology research during the period from 2020 to 2025 and their recurrences.

No.	Theory	Frequency	Key thinkers	Core idea	Representative references
1	Social justice theory	7	Rawls, Sen	Equity in resource distribution	Such as, Millard (2020)
2	Social development theory	5	Midgley, Freire	Linking economic and social progress	Such as, Sobantu (2020)
3	Collective capabilities	4	Sen, Nussbaum	Group-based capabilities	Such as, Burns (2022)
4	Sustainable development	3	Brundtland Commission	Environmental-social balance	Such as, McGregor and Scandrett (2022)
5	Critical development theory	3	Harvey, Foucault	Structural critique of development	Such as, Bolton (2022)
6	Social protection theory	2	Barrientos	Welfare and safety nets	Such as, Amoah (2020)
7	Social welfare theory	2	Nussbaum	Quality of life improvement	Such as, Sirén (2022)
8	Social dominance theory	2	Bourdieu	Power and inequality structures	Such as, Olonisakin and Idemudia (2021)
9	Neoliberalism critique	1	Harvey	Market-driven inequality critique	Gray, 2024
10	Social investment theory	1	Esping-Andersen	Human capital investment	Johnson, 2024
11	Psychological empowerment	1	Hong	Self-sufficiency and adaptation	Hong, 2024

In general, the theoretical trends addressed in recent global sociological works in development sociology research during the period from 2020 to 2025, as illustrated in Table 3, demonstrate researchers' interest in the theory's role in explaining development issues and topics related to their research. They also reflect the diversity and depth of their understanding of the social, economic, and political issues that impact development. For example, social justice theory, which is one of the main theoretical trends in these sociological studies, highlights the necessity of equality in the distribution of resources and opportunities, reflecting the need to promote social justice as a foundational element of sustainable development. This focus is considered a vital step toward addressing socio-economic gaps, particularly in the developing nations.

On the other hand, social development theory offers a conceptual framework that links economic growth with the improvement of social conditions, emphasizing the need to achieve a balance between economic and social factors. This trend reflects a growing recognition of the importance of social justice and equality in promoting development.

The collective capabilities theory, pioneered by Amartya Sen, highlights the importance of developing skills and capabilities within societies, enhancing individuals' ability to participate effectively in development. This interest by researchers reflects a shift from the traditional understanding that focuses on individuals to a more holistic understanding that considers social interaction.

It can be noted that, in light of the interest in these theoretical trends in global development sociology research, many theoretical approaches are likely to evolve to adapt to societal transformations in various development issues. For instance, critical theories of development are expected to evolve to include deeper analyses of global social, economic, and political relations. This development is expected to strengthen societies' capacities to resist dominance and pursue fair and inclusive development.

Overall, the future of global development sociology research seems to be shaped by a deeper understanding of the complex challenges facing societies, with a focus on issues of social justice, sustainability, and cooperation. These theoretical orientations are expected to form the basis for effective research aimed at promoting the wellbeing of individuals and communities in the future.

It is worth noting that, none of the prevailing theoretical frameworks identified in the reviewed studies directly addressed the social implications of AI, which also points to the need for theoretical innovation capable of accommodating the complexities of digital development.

### 6.3 Methodological approaches emphasized in global sociological works in development sociology research

The dominant approach in most global sociological works is the descriptive-analytical style. Only two studies, Niigaaniin et al. (2023) and Hong (2024) adopt an evaluative approach. Meanwhile, research styles such as exploratory, interpretive, and follow-up styles have completely disappeared, despite their big value in development sociology studies.

Global sociological studies are predominantly analytical and theoretical, at the expense of fieldwork. Approximately 47% of these studies were fieldwork, such as the work of Bautista et al. (2024), Cheng and Kumar (2022), and Amoah (2020). In contrast, approximately 53% of research papers have an analytical and theoretical nature, such as the papers by Rademacher and Pumar (2023), Fourie and Sands (2022), and the paper by Mohan (2022). This shows these works' awareness of the importance of fieldwork in studying development issues.

#### 6.3.1 Methodology

It is worth noting that the qualitative method, along with its tools, has significantly dominated global sociological research in development sociology. These works used various types of qualitative approaches, as some of them employed modern qualitative approaches such as, the research of Bautista et al. (2024), which relied on the Community-Engaged Research Method, which is used by scholars to understand and analyze social phenomena and issues related to development in local communities. This method requires researchers to interact with community members and groups through in-depth interviews, observations, focus groups, or even participation in daily community activities, with the necessity to be aware of the cultural, social, and economic context of the community (Amauchi et al., 2022, p. 3–20). Undoubtedly, such interactive qualitative approaches reflect a deeper understanding of development-related issues.

Table 4 shows the type and frequency of qualitative approaches used in global field-based sociological studies on contemporary development topics.

**Table 4:** Type and frequency of qualitative approaches used in global field-based sociological studies on contemporary development topics.

No.	Qualitative methodology	Frequency	Percent	No.
1	Case study	9	60%	6
2	Content analysis	4	26.6%	7
3	Participatory action research	1	6.8%	8
4	**Total field studies**	**15**	**100%**	9

Bold values indicate the total frequencies for each category.

The data given in Table 4 shows the predominance of the case study approach as a qualitative approach in sociological fieldwork of a qualitative nature, accounting for 60% of all global work. This confirms the effectiveness of these works in providing insight, depth, and, subsequently, interpretation of the development issues they studied. As for the methods used in these quantitative sociological fieldworks, the social survey approach was the only approach used in a single study—Olonisakin and Idemudia (2021). This indicates the significant reliance of the global sociological fieldwork in development sociology research on qualitative approaches.

This indicates that the Methodological Trends are:

- Qualitative dominance (case studies, interviews, focus groups).
- Limited quantitative research.
- Minimal computational/AI-based methods.

The reviewed studies were characterized by a predominance of qualitative methodologies, while quantitative and computational methods appeared less frequently in the selected studies.

#### 6.3.2 Data collection tools

These works used qualitative tools, as interviews topped the list of most frequently used data collection tools, such as in Pankaj and Yadav (2023), followed by focus groups Amoah (2020), and direct observation, Galán-Guevara (2024). As for quantitative data collection tools, the questionnaire was used only once in the study by Olonisakin and Idemudia (2021).

#### 6.3.3 Sample

Purposeful sampling was the most commonly used, appearing in 13 sociological works, such as Hong (2024). In contrast random sampling method was used in only two works, such as that by Olonisakin and Idemudia (2021).

Sampling units used in global sociological works in development sociology research varied and included development programs beneficiaries, workers of development institutions, development program researchers, members of funeral insurance, families, local organizations, and social development conferences.

In general, the preceding methodologies overview, data collection tools, and sampling methods in contemporary developmental sociology research illustrates that these methods remain conservative and fail to take advantage of AI techniques, such as computational methods, machine learning techniques, and big data analytics, despite the growing importance of AI-enhanced social research.

### 6.4 PRISMA-based interpretation

The PRISMA-guided screening process resulted in a systematic narrowing of the initial 112 identified records to 35 final studies. This reflects rigorous criteria for subject-matter significance but also highlights a relatively narrow empirical base in the global sociology of development literature.

### 6.5 The AI integration gap

A significant finding is the limited integration of AI into the sociology of development research. Most studies address AI indirectly, rather than considering it as a methodological or analytical tool, indicating a clear research gap within the frameworks of computational sociology and digital development.

Despite the limited references to AI in the reviewed literature, a more detailed analysis reveals several emerging trends in its understanding within the sociology of development research. Some studies have described AI as a transformative technological force capable of reshaping social welfare systems, labor markets, and governance mechanisms, thereby contributing to broader processes of social change. In contrast, other studies have adopted a critical perspective, highlighting the challenges associated with algorithmic bias, digital exclusion, and the potential for exacerbating existing social and structural inequalities. Additionally, a limited number of studies have addressed AI as a methodological tool capable of enhancing evidence-based decision-making and facilitating data-driven strategies for development planning and policy formulation.

## 7 Limitations of the study

Although this review provides a comprehensive overview of global research trends in the sociology of development between 2020 and 2025, some limitations should be acknowledged. First, the review was limited to studies indexed in Scopus, SAGE, Wiley, and GISTOR databases, which may have excluded relevant publications available in other databases or unpublished literature sources. Second, the analysis focused exclusively on studies published during the 2020–2025 period; therefore, the findings may not fully reflect previous theoretical developments or future shifts in the field. Furthermore, the study approached AI as a broad analytical category encompassing diverse technological developments. Given the rapid evolution of AI during the 2020–2025 period, particularly the emergence of generative AI and big language models, the review did not systematically differentiate between specific AI subfields. Future research may benefit from investigating how different forms of AI impact development processes and social research, potentially leading to more nuanced interpretations of the relationship between AI and the sociology of development. Third, in keeping with the nature of scope reviews, the study aimed to map and compile existing literature rather than conduct a detailed assessment of the methodological quality of individual studies.

An additional limitation of this review is its focus on studies explicitly falling within the field sociology of development and meeting predefined inclusion criteria. Consequently, the review may not have encompassed interdisciplinary research addressing the intersection of artificial intelligence and development from broader economic, environmental, or technological perspectives. Therefore, findings regarding the limited integration of AI should be interpreted within the context of the reviewed studies, rather than reflecting the entirety of the existing scholarly landscape.

Finally, the relatively limited number of studies explicitly addressing the intersection of the sociology of development and AI has limited the possibility of drawing more comprehensive conclusions about the role of AI in this field. These limitations also point to important directions for future research aimed at expanding and deepening academic understanding of this emerging field.

## 8 Conclusion and future directions

This scoping review achieved its objectives through identifying the prevailing thematic priorities, theoretical perspectives, and methodological approaches that characterize development sociology research between 2020 and 2025. The findings indicate that contemporary sociology of development continues to highlight social justice, sustainable development, social policies, and community participation, reflecting the discipline's enduring interest in inequality and social transformation. The review also reveals increasing theoretical diversity and methodological pluralism, particularly through the continued use of qualitative and participatory approaches.

However, the analysis also reveals a significant gap between the sociology of development and the rapidly evolving AI field. While AI has become a transformative force shaping social institutions, labor markets, governance systems, and development practices worldwide, its integration into the sociology of development remains limited. In most of the reviewed studies, AI appeared only indirectly or as a contextual factor, rather than as a central analytical category. This finding suggests that the discipline has not fully adapted its conceptual and methodological tools to address emerging forms of inequality mediated by digital technologies, algorithmic governance, and technology-driven social change.

From a critical perspective, these findings raise important questions about the future relevance of the sociology of development. Its ability to explain contemporary development processes increasingly depends on its capacity to engage with technological transformations while maintaining its traditional commitment to social justice, participation, and human-centered development. Failure to integrate these dimensions may limit its explanatory power in understanding the complexities of twenty-first-century development.

Accordingly, future research should move beyond viewing AI as merely a technological innovation and instead address it as a social phenomenon with profound developmental implications. This requires multidisciplinary collaboration between sociology, data science, and policy studies, alongside the development of ethical frameworks that ensure technological advancements contribute to inclusive, equitable, and sustainable development outcomes.

Based on the findings of this review, several recommendations can be made to advance research in development sociology. First, future studies should foster interdisciplinary collaboration by integrating sociological perspectives with data science and AI to better address emerging development challenges. Second, researchers should continue adopting methodological pluralism, combining qualitative insights with quantitative and computational methods to understand the complexity of contemporary development processes. Third, greater attention should be paid to underexplored issues, including the sociological implications of digital transformation, algorithmic inequality, and the role of civic engagement in sustainable development. Finally, the development sociology should prioritize ethical frameworks that promote social justice and inclusivity, as well as human-centered approaches, when examining AI applications in development.

## Data Availability

The original contributions presented in the study are included in the article/supplementary material, further inquiries can be directed to the corresponding author.
